# Management of Terson Syndrome: Long-Term Experience in a Single Center

**DOI:** 10.3390/biomedicines12102336

**Published:** 2024-10-14

**Authors:** Angelo Maria Minnella, Martina Maceroni, Carmela Grazia Caputo, Paola Sasso, Gabriele Verardi, Danio De Simone, Gabriele Ciasca, Stanislao Rizzo, Maria Gabriella Buzzi, Cecilia Della Vedova, Rita Formisano

**Affiliations:** 1 Istituto di Oftalmologia, Università Cattolica del Sacro Cuore, 00168 Rome, Italy; angelomaria.minnella@unicatt.it (A.M.M.); gabrver@hotmail.it (G.V.); danio.desimone01@icatt.it (D.D.S.); stanislao.rizzo@unicatt.it (S.R.); 2 Fondazione Policlinico Universitario A. Gemelli—IRCCS, 00168 Rome, Italy; carmelagrazia.caputo@gmail.com (C.G.C.); paoladottsasso@gmail.com (P.S.); gabriele.ciasca@unicatt.it (G.C.); 3 Dipartimento di Neuroscienze, Sezione di Fisica, Università Cattolica del Sacro Cuore, 00168 Rome, Italy; 4 Fondazione Santa Lucia—IRCCS, 00142 Rome, Italy; mg.buzzi@hsantalucia.it (M.G.B.); c.dellavedova@hsantalucia.it (C.D.V.); r.formisano@hsantalucia.it (R.F.)

**Keywords:** Terson Syndrome, intraocular hemorrhage, subarachnoid hemorrhage, vitreous hemorrhage, vitrectomy

## Abstract

Background/Objectives: Terson Syndrome (TS) is a rare entity consisting of an intraocular hemorrhage secondary to subarachnoid hemorrhage (SAH) or intracerebral hemorrhage (IH). This study aimed to retrospectively describe the experience of the Ophthalmology Unit of Policlinico Gemelli, Rome, in the management of TS. Methods: Twenty-four eyes of 19 patients (10 males—53%; 9 females—47%; mean age of 44.73 ± 12.49 years) with TS who had pars plana vitrectomy between 2011 and 2024 were included. The primary outcome was the mean change in best-corrected visual acuity (BCVA) 1–3 months after surgery. The secondary outcome was the correlation of post-operative BCVA with the timing of vitrectomy (early vs. late, ≤100 or >100 days). Results: The time between diagnosis and surgery ranged from 33 to 284 days (median = 102 days, interquartile range IQR = 74–161). Baseline BCVA ranged from 6 to 50 ETDRS letters with a median of 17 letters (IQR = 15–25) and significantly increased after surgery, with a median value of 80 (IQR = 70–85). The BCVA percentage improvement had a median of 325% (IQR = 300–431%). No differences in post-operative BCVA were found between patients who underwent early or late vitrectomy. One vitrectomy was complicated by an endophthalmitis. Conclusions: Although no clear guidelines exist on managing TS, vitrectomy significantly improves BCVA, and a delay in surgical intervention does not necessarily worsen the functional outcome. However, an early vitrectomy could improve the stimuli perception, facilitating the rehabilitation process.

## 1. Introduction

Terson Syndrome (TS) incorporates various forms of ocular hemorrhage, including retinal, preretinal, and vitreous hemorrhage, resulting from acute intracranial hemorrhage. A recent study estimated the incidence of TS to be 21% after subarachnoid hemorrhage (SAH) when the true incidence of SAH in the United States is 30,000 new cases per year [1,2,3]. These data on TS likely underestimate the real incidence, because of the significant mortality after intracranial hemorrhagic events. However, TS can be considered a rare entity. A recent multicenter Canadian study reporting the 10 years-experience of three Ophthalmological Centers on TS, described the pre-operative characteristics and the post-vitrectomy outcomes of 14 eyes of 11 patients [1]. Other less recent studies reporting ophthalmological data on patients with TS are only small case series [4,5,6,7].

Considering the scarce literature evidence, we decided to report the long-term experience of our center in managing TS, having a significant cohort of subjects for such a rare disease. The main outcome of the study was the change in best-corrected visual acuity (BCVA) from baseline (pre-operative visit) to 1–3 months after vitrectomy (post-operative visit). The secondary outcome was the correlation of post-operative BCVA with the timing of vitrectomy (early vs. late, ≤ or >100 days after intracranial hemorrhage). Potential correlations between demographical and clinical variables and the surgical outcome were also investigated.

## 2. Materials and Methods

This retrospective study adhered to the Declaration of Helsinki and was approved by the ethics committee of the Fondazione Policlinico Universitario A.Gemelli, Rome. The analysis was conducted on 24 eyes of 19 consecutive patients with intraocular hemorrhage secondary to intracranial hemorrhage, regardless of the mechanism, who were referred to the Ophthalmology Unit of Policlinico Gemelli between 2011 and 2024, both from the Intensive Care Unit (ICU) of Policlinico Gemelli, Rome, and from the Neuromotor Rehabilitation Institute “Santa Lucia Foundation”, IRCCS.

All the patients underwent pre-operative ocular B-scan ultrasonography and standard small-gauge pars plana vitrectomy. Clinical and ophthalmological data were retrospectively evaluated. Specifically, sex, age at diagnosis, cause of intracranial hemorrhage, the ultrasonographic pattern of the intraocular hemorrhage, baseline best-corrected visual acuity (BCVA) when available, the period between presentation and vitrectomy, surgical procedures, any surgical complication, and post-operative BCVA (when available) were collected. Data were extracted from the patients’ medical charts and collected in Excel files.

Statistical analyses of the data and visualization were conducted using the OriginPro2022 software tool. Categorical variables were represented as counts or percentages. Continuous variables were tested for normality using the Shapiro–Wilk test and qualitative observation of QQ plots (data not reported). In some variables, including pre- and post-operative BCVA and their difference, statistically significant deviations from normality were found. Therefore, continuous variables were discussed in terms of median and interquartile range (IQR), reporting the first quartile (Q1) and the third quartile (Q3) as follows (Q1–Q3). The comparison between pre- and post-operative BCVA values was conducted using a Wilcoxon–Mann–Whitney test for paired data. Statistical significance was set at 0.05. The correlation between variables was studied by calculating Spearman’s correlation coefficient, and the data were arranged in a correlation plot, using a dual color code to indicate the intensity and the sign of the correlations.

## 3. Results

**Figure 1:**
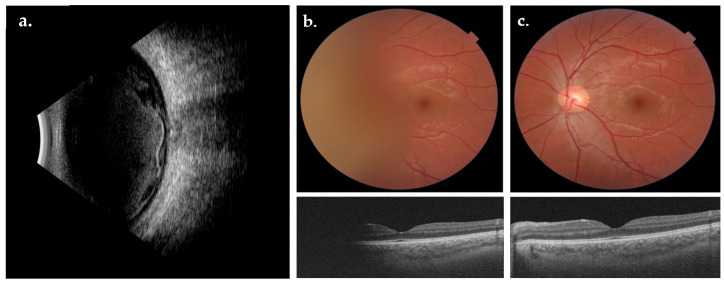
B-scan ultrasonography of a case of Terson Syndrome (**a**). Pre-operative fundus photograph (**b**, **top**) showing a vitreous hemorrhage anterior to the optic disc and pre-operative macular scan (**b**, **bottom**). One-month post-operative fundus photograph (**c**, **top**) and OCT macular scan (**c**, **bottom**).

**Figure 2:**
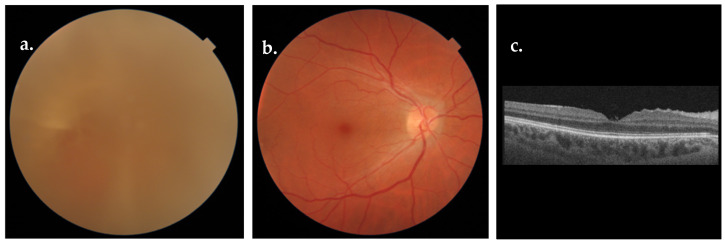
Pre-operative fundus photograph (**a**), post-operative fundus photograph (**b**), and OCT macular scan (**c**).

Twenty-four eyes of 19 patients were included in the study: 10 patients were male (53%) and 9 were female (47%), with a median age of 49 years at baseline (IQR = 41–51 years). Among the included eyes, 15 were right eyes, whereas 9 were left eyes. All the examined eyes showed a similar pattern at B scan ultrasonography consisting of the presence of a large amount of intraocular hemorrhage anteriorly to the optic disc and macular region, often with a modification of the internal limiting membrane (ILM) due to sub-ILM blood dispersion (Figure 1). Pre-operative fundus examination revealed a complete or partial vitreous hemorrhage in all cases. The visualization of macular scans at Optical Coherence Tomography (OCT) was totally or partially blocked by vitreous hemorrhage in most cases (Figure 1). All 24 eyes included underwent surgical treatment (Figure 2).

Five out of nineteen patients presented a bilateral involvement, albeit asymmetrical, and vitrectomy was performed on both eyes, with a mean interval of 47 days between the two eyes (range 24–82). Surgery was performed by a single surgeon (A.M.M.). Specifically, the 23G and the 25G technique were used in three and twenty-one cases, respectively.

Different types of vitreal tamponades were used after vitreous removal: in particular, a Balanced Saline Solution (BSS)/Air exchange was used in twenty-one eyes, a BSS/Air/SF6 exchange was used in two eyes, and a BSS/Air/C3F8 exchange was used in one eye. One patient underwent vitrectomy, cryotherapy, and endolaser for the presence of a retinal hole. One other patient had endolaser for a retinal hole.

All the clinical and ophthalmological data are summarized in Table 1, including the pre- and post-surgery BCVA. In some cases, BCVA values were not easily accessible due to neurological impairment. However, baseline BCVA ranged from 6 to 50 ETDRS letters when evaluable, with a median value of 17 letters (IQR =15–25 letters).

**Figure 3:**
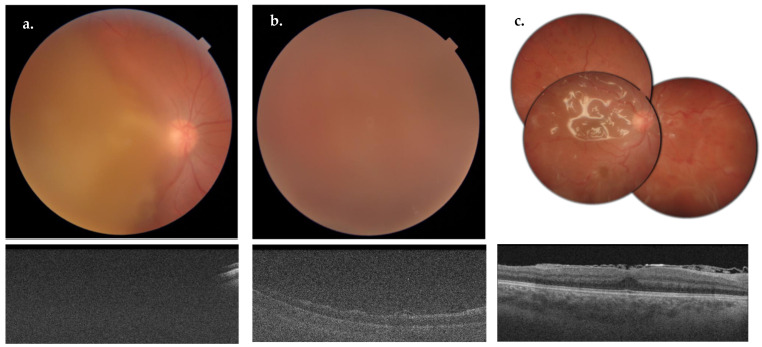
Pre-operative fundus photograph (**a**, **top**) and OCT macular scan (**a**, **bottom**) of the case 12-RE. Fundus photograph (**b**, **top**) and OCT macular scan (**b**, **bottom**) showing a post-operative endophthalmitis. Multiple fundus photographs showing diffuse dot-blot hemorrhages and silicon oil in the vitreous cavity (**c**, **top**) after 25 G vitrectomy for endophthalmitis. OCT macular scan (**c**, **bottom**) showing a substantially preserved macular anatomy, with evidence of silicon oil in the vitreous cavity.

A variable amount of time was reported between the acute brain event and the surgery, depending on the different patients’ conditions, with a median of 102 days (IQR = 74–161 days). Only one patient presented a post-operative complication: after 4 days from surgery, an endophthalmitis was diagnosed (Figure 3). Phacoemulsification and a second vitrectomy with epiretinal membranes peeling and silicone oil tamponage were performed (Figure 3). After 8 weeks, silicon oil was removed and an intraocular lens was implanted in the sulcus.

**Figure 4:**
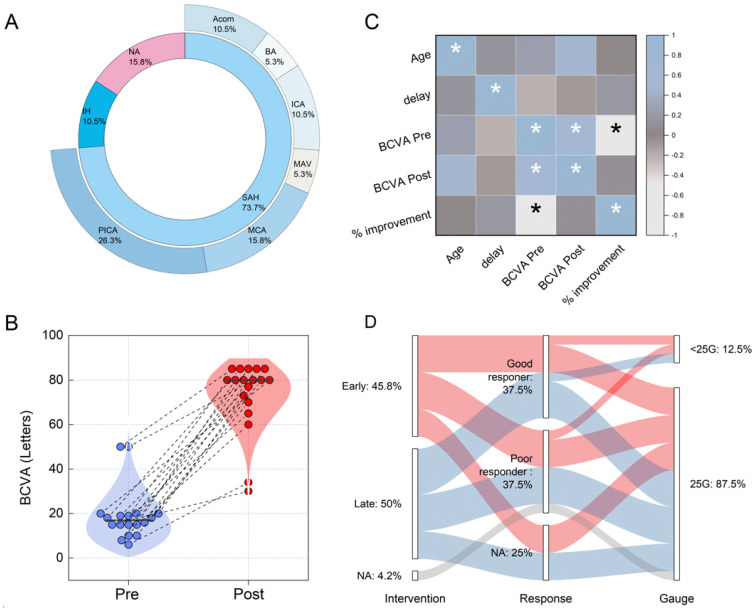
(**A**) Sunburst plot illustrating the primary causes of intraocular hemorrhage within the inner circle and detailing the arterial locations or regions involved in subarachnoid hemorrhage (SAH) in the outer circle. (**B**) Visualization of changes in best-corrected visual acuity (BCVA) from pre- vitrectomy (blue dots) to post-vitrectomy (red dots) for each patient, with dashed lines connecting pre- and post-surgery values to highlight individual changes. (**C**) Correlation matrix employing Spearman’s coefficients to analyze the relationships among patient age, time delay to surgery, and BCVA changes before and after surgery. Asterisks (*) denote statistically significant correlations (*p* < 0.05). (**D**) Alluvial plot showing the timing of surgical intervention (early vs. late) linked to patient outcomes, categorized by treatment response into ‘good responders’ (those whose BCVA improvement was greater than or equal to the median increase of 325%) and ‘poor responders’ (those below this threshold). The plot also distinguishes the surgical gauge used, noting whether it was smaller or equal to 25 G, to explore the impact of instrument size on surgical outcomes.

The intraocular hemorrhage was associated in fourteen patients (74%) with a spontaneous subarachnoid hemorrhage (SAH) due to a cerebral aneurysm: the most common locations of the cerebral aneurysm were the posterior inferior cerebellar artery (n = 5; PICA), medial cerebral artery (n = 3; MCA), anterior communicating artery (n = 2; Acom), internal carotid artery (n = 2; ICA), and basilar artery (n = 1; BA). In one (5%) patient, it was caused by an arterial–venous malformation (MAV). In two (11%) patients, the intraocular hemorrhage was secondary to head trauma. In the remaining patients, data on the cause of the intracranial hemorrhage were not available. Data regarding the cause and location of TS in the recruited subjects are summarized in Figure 4A.

The median post-operative BCVA was 80 ETDRS letters (IQR = 70–85 letters). A comparison of the pre- and post-vitrectomy BCVA values is depicted in Figure 4B, where data for each operated eye at the two different time points are connected by dashed lines. A visual analysis of Figure 4B indicates a significant increase in BCVA following the surgery across all points. The results from a Wilcoxon signed-rank test for paired data demonstrate a highly statistically significant increase in BCVA (*p* = 3.86 × 10^−6^). The median increase in BCVA for the recruited patients was estimated at 59 ETDRS letters (IQR = 45–67 letters). The percentage improvement was also calculated using the formula $\%Improvement=\left({BCVA}_{Post}-{BCVA}_{Pre}/{BCVA}_{Pre}\right)\times 100$. This calculation revealed a median value of 325% (IQR = 300–431%).

Figure 4C explores correlations among clinical variables including patient age, the time elapsed between the acute event and the surgery (referred to as ‘delay’), and the pre- and post-surgery BCVA values, along with the percentage increase. The data are presented in a correlation map using Spearman’s correlation coefficients, according to material and methods section. An asterisk marks statistically significant correlations (*p* < 0.05). Interestingly, there are no statistically significant correlations between age and BCVA values before or after surgery, nor with the percentage improvement. These variables also do not correlate significantly with the time between the acute event and the intervention, suggesting that a delayed intervention does not necessarily worsen outcomes. The significant correlation between pre-surgery BCVA and percentage increase, shown in Figure 4C, is not further discussed, as it stems from the mathematical definition of the latter.

To further investigate the potential role of delay time, we divided our cohort into two groups: early intervention (≤100 days) and late intervention (>100 days). The analysis revealed no statistically significant differences between these two groups in terms of percentage improvement (U = 59, *p* = 0.68) or absolute improvement in BCVA (U = 37, *p* = 0.96), according to the Wilcoxon–Mann–Whitney U test.

The absence of correlation between the timing of the intervention (early or late) and the outcomes is further illustrated in Figure 4D, where we present an alluvial plot that connects the timing of the intervention with patient outcomes. For this analysis, patients were further categorized as ‘good responders’ or ‘poor responders’ based on whether their percentage improvement was greater than or equal to the median value of 325%. Data not available are represented as a separate category, marked ‘NA’. The plot also explores the relationship between the surgical technique used, particularly concerning the gauge value, and the outcomes of the surgery. Here again, no clear patterns are discernible among the data.

Ten out of the nineteen enrolled patients showed a neurological and neuropsychological improvement after surgery, especially in the collaboration to the neurorehabilitation program.

## 4. Discussion

This study aimed to report the 14-year experience of a single center in managing TS. Considering that no clear guidelines exist on the management of this condition, we retrospectively analyzed the data of 19 patients affected by TS who underwent vitreal surgery, trying to define a standardized and optimized approach to this rare entity. As expected, in our sample, post-operative BCVA significantly improved after surgery. The assessment of pre-operative BCVA is often limited in patients with TS, given the presence of concomitant neurological deficits. In accordance with a recent study [1], no correlation was found between post-operative BCVA and the timing of vitrectomy. The post-operative functional outcome did not differ between patients who had surgery ≤100 days after the intracranial event and patients who had surgery >100 days after the intracranial event. Similar results were reported by Narayanan et al., who revealed no significant difference in final BCVA between patients who received early vitrectomy for a traumatic brain injury-induced vitreous hemorrhage compared to the late-vitrectomy group (surgery after 3 months) [8]. We chose the 100-day cut-off because the literature evidence suggests that it can represent an adequate amount of time for observational therapy. The observational time for at least 3 months before offering a vitrectomy can optimize the risk–benefit ratio, especially if the partner eye allows spatial orientation [9].

Therefore, it could be concluded that ocular hemorrhaging in TS could be managed conservatively for spontaneous improvement without the risk of reduced visual potential.

However, we have some considerations. Although patients with TS are often severely neurologically impaired, vitrectomy rapidly improves the perception of visual stimuli, enhancing the connection with the environment and the rehabilitation process. The beneficial effects of a timely vitrectomy are particularly evident in bilateral cases. The safety and efficacy of small gauge, sutureless surgery has indeed a profile that invites earlier intervention. In addition, long-standing vitreous hemorrhages can be more challenging for the surgeon. The blood can assume a cretaceous consistency, making vitrectomy more difficult and increasing the risk of post-operative complications. In some cases, endovitreal clot removal can require instruments with a larger caliber, with an increased risk of retinal tractions and retinal detachment. Moreover, different studies found that delayed surgery might be complicated by an epiretinal membrane, retinal detachment, and macular degeneration [4]. According to Garweg et al. [9], minimizing the latency before surgery is associated with better outcomes (BCVA of 0.87 ± 0.27 for those operated within 90 days versus 0.66 ± 0.31 for those vitrectomized after 3 months). Although the optimal timing of surgery remains still uncertain, other authors advocate for early vitrectomy, with a mean duration of <6 months suggested to be effective [10,11,12,13]. Supporting the need for a timely vitrectomy, there is evidence that the breakdown products of intraocular blood had toxic effects on the animal retina: the fibrin could attach to the photoreceptor 25 min after bleeding and exert toxic action within 1 h [14]. In our sample, the eye with the longest interval between SAH and vitrectomy (case 8) showed a poor post-operative visual outcome, although the surgery was uncomplicated.

A late diagnosis is probably the main cause of delayed surgery. For this reason, a screening with fundus examination enables a faster diagnosis of TS in all patients with intracranial vascular events. A recent study proposed ocular ultrasonography with standard equipment as a reliable, easy-to-handle bedside screening tool for detecting TS [15]. However, a dilated fundus examination remains the easiest procedure to detect TS.

The strength of this study is represented by the longitudinal data collection approach, able to provide long-term outcomes in patients with SAH. The limitations of the study are (i) the small number of enrolled patients; (ii) the lack of neurological and neuropsychological evaluation scales before and after survey; and (iii) the lack of long-term outcome follow-up.

## 5. Conclusions

In conclusion, vitrectomy significantly improves BCVA in patients with TS. Delay in surgical intervention is not necessarily correlated with a worsening of the functional outcome. However, considering the evident beneficial effect of surgery, an early vitrectomy could ameliorate the perception of stimuli and the rehabilitation process (Box 1). Further studies with larger samples would be needed to better define the optimized approach to this rare entity and the impact of surgery on the neurological and neuropsychological final outcome of the patients with severe acquired brain injury.

Box 1 **Summarizes all the relevant findings of the study and offers clinical pearls for specialists.**

**Clinical pearls**

- Every patient with intracranial vascular events should undergo fundoscopic examination for the diagnosis of Terson Syndrome (TS).
- Vitrectomy significantly improves BCVA in patients with TS; a delay in surgical intervention does not necessarily worsen the functional outcome.
- However, an early vitrectomy could improve the stimuli perception, facilitating the rehabilitation process.

## Figures and Tables

**Table 1:** Clinical and ophthalmological data.

Case	Sex	Age	Type of Intracranial Hemorrhage (Source of Hemorrhage)	Type of Vitrectomy	Days between Cerebral Event and Vitrectomy	Pre-Operative BCVA—ETDRS Letters	Post-Operative BCVA—ETDRS Letters
**1**	F	46	IH and SAH due to a cerebral aneurysm (right MCA)				
RE				25G	53	20	85
**2**	F	49	SAH due to a cerebral aneurysm (left PICA)				
RE				25G	100	50	80
LE				25G	72	8	80
**3**	F	53	SAH due to a cerebral aneurysm (left PICA)				
RE				25G	207	16	85
LE				25G	157	18	85
**4**	M	41	SAH due to a cerebral aneurysm (Acom)				
RE				25G	71	15	73
**5**	F	49	SAH due to a cerebral aneurysm (right MCA)				
RE				25G	217	NA	NA
**6**			SAH due to a cerebral aneurysm (left ICA)				
LE	F	49		25G	33	NA	NA
**7**	M	49	SAH due to a cerebral aneurysm (left PICA)				
RE				23G	79	10	80
LE				23G	161	10	80
**8**	M	24	SAH due to a MAV (NA)				
RE				25G	284	6	34
**9**	F	43	SAH due to a cerebral aneurysm (NA)				
LE				25G	60	50	85
**10**	M	31	IH due to a cranic trauma (intraparenchymal)				
LE				25G	56	19	80
**11**	F	66	SAH due to a cerebral aneurysm (right ICA)				
RE				25G	205	20	80
**12**	M	18	IH due to trauma (NA)				
RE				25G	100	15	65
LE				25G	128	20	85
**13**	M	49	SAH due to a cerebral aneurysm (left PICA)				
RE				25G	123	NA	NA
LE				25G	177	NA	NA
**14**	F	45	SAH due to a cerebral aneurysm (left PICA)				
RE				25G	109	15	60
**15**			SAH due to a cerebral aneurysm (right MCA)				
RE	M	46		23G	86	15	30
**16**	M	56	Idiopathic IH (intraparenchymal)				
LE				25G	90	NA	NA
**17**	M	28	SAH due to a cerebral aneurysm (Acom)				
RE				25G	100	18	70
**18**	F	57	SAH due to a cerebral aneurysm (BA)				
RE				25G	100	NA	NA
**19**	M	51	NA				
RE				25G	NA	19	77

Abbreviations: SAH, subarachnoid hemorrhage; IH, intracerebral hemorrhage; PICA, posterior inferior cerebellar artery; MCA, medial cerebral artery; Acom, anterior communicating artery; ICA, internal carotid artery; BA, basilar artery; NA, not available; BCVA, best-corrected visual acuity; MAV, arterial–venous malformation; RE, right eye; LE, left eye.

## Data Availability

Data are contained within the article.

## List of Abbreviations (Alphabetical Order)

Anterior communicating artery (Acom), arterial–venous malformation (MAV), Balanced Saline Solution (BSS), basilar artery (BA), best-corrected visual acuity (BCVA), internal carotid artery (ICA), Intensive Care Unit (ICU), internal limiting membrane (ILM), interquartile range (IQR), intracerebral hemorrhage (IH), left eye (LE), medial cerebral artery (MCA), not available (NA), Optical Coherence Tomography (OCT), posterior inferior cerebellar artery (PICA), right eye (RE), subarachnoid hemorrhage (SAH), Terson Syndrome (TS).
